# Between Life and Ethics: A Case of Extreme Azotemia and Decision-Making Challenges

**DOI:** 10.51894/001c.143753

**Published:** 2025-09-30

**Authors:** Sofia Tiberio, Alex Pasculle, Harsh Patel, Rajita Ramaraju, Jonathan Lopez

**Affiliations:** 1 College of Osteopathic Medicine Michigan State University; 2 College of Osteopathic Medicine Michigan State University; 3 Medical Intensive Care Henry Ford Macomb

## 129

### INTRODUCTION

Severe azotemia is typically associated with profound neurological impairment and multisystem dysfunction. We present a case of a patient with the highest recorded Blood Urea Nitrogen (BUN) level of 285 mg/dL who remained neurologically intact until the terminal event. This case underscores the clinical variability of azotemia and highlights the challenges in medical decision-making and guardianship in critically ill patients.

### CASE PRESENTATION

A 53-year-old male with a history of hypertension and medical noncompliance presented with dyspnea, bilateral lower extremity edema, and abnormal ECG findings. Initial laboratory studies demonstrated acute kidney injury with a BUN of 205 mg/dL and creatinine of 42.3 mg/dL. Despite the severity of his azotemia, the patient remained alert and oriented, with minimal uremic symptoms. Nephrology and intensive care teams recommended emergent renal replacement therapy (RRT), which the patient repeatedly declined.

Concerns regarding his decision-making capacity prompted psychiatric and ethics consultations. While initially deemed competent, his persistent refusal of life-sustaining interventions led to a reassessment, ultimately determining that he lacked capacity. Emergency guardianship was pursued; however, the process was delayed, prolonging his lack of definitive treatment. During this period, his renal function continued to deteriorate, with BUN rising to 285 mg/dL and creatinine to 54.87 mg/dL. Despite severe metabolic derangements, he remained neurologically intact. On hospital day 17, he developed bradycardia and hyperkalemia (8.1 mEq/L), culminating in torsades de pointes and subsequent asystole. His documented DNR status was upheld, and resuscitative efforts were not initiated.

### DISCUSSION

This case represents the highest reported BUN in a living patient, challenging the presumed correlation between azotemia severity and neurological impairment. It also highlights the limitations in assessing decision-making capacity in critically ill patients and the logistical hurdles in obtaining emergency guardianship for timely intervention.

### CONCLUSION

This case underscores the unpredictable clinical manifestations of extreme azotemia and the ethical complexities of managing patients who refuse life-sustaining therapy. It emphasizes the need for streamlined protocols in evaluating medical decision-making capacity and expediting emergency guardianship in critically ill patients.

